# Optimizing Surgical Performance: Evidence-Based Feedback Strategies for Trainees and Junior Staff

**DOI:** 10.7759/cureus.111316

**Published:** 2026-06-22

**Authors:** David Heath, Kyungchul Kim, Amyn G Pardhan, Jacinta Cover

**Affiliations:** 1 General Surgery, Bunbury Regional Hospital, Perth, AUS; 2 General Surgery, Fiona Stanley Hospital, Perth, AUS; 3 General Surgery, Bunbury Regional Hospital, Bunbury, AUS; 4 General Surgery, Western Australian Country Health Service, Bunbury, AUS

**Keywords:** competency-based education, educational feedback, feedback models, performance assessment, postgraduate training, racs, simulation training, surgical education

## Abstract

Background: Feedback is fundamental to surgical training, underpinning skill acquisition, clinical decision-making, and the development of safe, competent surgeons. Despite its recognised importance, the consistent delivery of high-quality, effective feedback remains a significant challenge within busy clinical environments, including those in Australia and New Zealand (ANZ). This paper synthesises current evidence to provide practical guidance for surgical educators and trainees.

Methods/Approach: A literature review was conducted using major biomedical databases (PubMed/MEDLINE, Embase) focusing on feedback in postgraduate surgical education. Search terms included "feedback," "surgical training," "surgical education," "assessment," "performance," and specific feedback models. The review synthesised evidence on feedback models, effectiveness, implementation challenges, best practices, the role of technology, and factors pertinent to the ANZ context.

Results/Key Principles: Effective feedback appears to be timely, specific, based on direct observation, objective, actionable, and delivered respectfully within a supportive relationship. While various models exist (e.g., Pendleton, SET-GO (What I Saw, what Else did I see, what Thinking was involved, what Goals arise, Offers/Opportunities)), their optimal application depends on context and goals. Key challenges include time constraints, inconsistent supervisor skills, inadequate observation, and cultural factors impacting feedback exchange. Best practices involve establishing psychological safety, collaborative goal setting, and focusing on behaviour. Simulation and video review offer valuable adjuncts for feedback delivery outside the operating theatre. Specific considerations for ANZ include the The Royal Australasian College of Surgeons (RACS) training structure and geographical diversity.

Conclusion: Optimising feedback in surgical training requires a multifaceted approach, moving beyond ad-hoc comments towards structured, evidence-based practices. Implementing established principles, training supervisors, leveraging technology, and fostering supportive institutional cultures are crucial steps for enhancing surgical performance and patient safety in the Australasian setting and potentially internationally. This synthesis provides actionable strategies for surgeons, trainees, and educators committed to improving surgical education outcomes.

## Introduction and background

The imperative of effective feedback in surgical training

The development of surgical competence is a complex process reliant on acquiring technical proficiency, sound clinical judgement, effective communication, and professionalism [1,2]. Central to this journey is the iterative cycle of performance, observation, reflection, and guidance. Within this cycle, feedback serves as the critical catalyst, transforming experience into learning and driving improvement [3,4]. High-quality feedback, defined as specific, timely, and actionable information provided to a learner about their performance, is not merely beneficial; it is essential for trainees to understand their strengths, identify areas for development, and calibrate their self-assessment against expert expectations [5,6]. Its impact extends beyond technical skill acquisition; effective feedback cultivates reflective practice, enhances clinical reasoning, supports professional identity formation, and ultimately contributes to improved patient safety outcomes [7,8].

Current landscape and challenges

Despite widespread agreement on its importance, the provision of effective feedback in surgical training environments faces persistent challenges globally, including within Australia and New Zealand (ANZ) [9,10]. The high-stakes, time-pressured nature of clinical surgery often relegates feedback to brief, informal comments, frequently delivered inconsistently or omitted entirely [11]. Common barriers reported in the literature include significant time constraints for both supervisors and trainees, lack of direct observation of critical performance aspects, variability in supervisors’ feedback skills and confidence, and logistical difficulties in finding appropriate time and space for meaningful discussion [12,13]. Furthermore, traditional hierarchical structures prevalent in surgery can create psychological barriers, making trainees hesitant to reveal perceived weaknesses or seek clarification, and supervisors potentially reluctant to provide constructive criticism for fear of damaging rapport or provoking defensiveness [14]. The result is often feedback that is perceived by trainees as too vague, infrequent, subjective, or overly focused on negative aspects, limiting its educational value [15]. Addressing these practical constraints and cultural factors is paramount, as simply advocating for more feedback without acknowledging the context in which supervisors operate is unlikely to yield substantial change [16].

The Australasian context

Surgical training in ANZ, primarily overseen by the Royal Australasian College of Surgeons (RACS), presents a unique context [17]. The structured nature of RACS training programmes, the geographical diversity spanning major metropolitan centres to remote and rural locations, and the specific cultural milieu influence how feedback is delivered and received [18]. While national standards exist, variability in feedback practices across different training sites and specialties likely persists. Recent attention to competency-based medical education and non-technical skills, such as leadership and communication, further underscores the need for robust feedback mechanisms that address the full spectrum of surgical competence within this specific system [19,20]. Understanding any unique local challenges or successful initiatives, potentially identified through research conducted by prominent ANZ surgical education researchers and institutions, is crucial for tailoring effective strategies.

Rationale and objectives

Given the critical role of feedback and the persistent challenges in its implementation, particularly within the demanding surgical field, there is a need for clear, evidence-based guidance for both supervisors (consultant surgeons, fellows) and trainees (registrars, junior doctors) in the ANZ setting. This paper aims to synthesise the current evidence on effective feedback mechanisms applicable to postgraduate surgical training. Specifically, this review identifies the core principles of effective feedback based on educational theory and empirical research, describes and evaluates established and contemporary feedback models, summarises the evidence regarding the effectiveness of various feedback techniques, discusses common challenges and best practices for feedback delivery in clinical surgical settings, explores the role of simulation and technology in facilitating feedback, and provides recommendations tailored for implementation within the ANZ healthcare system and RACS training programmes, relevant across diverse surgical specialties. The ultimate goal is to provide a resource that supports the continuing education of surgeons and enhances the training experience for the next generation. Given the critical role of feedback and the persistent challenges in its implementation, particularly within the demanding surgical field, there is a need for clear, evidence-based guidance for both supervisors (consultant surgeons, fellows) and trainees (registrars, junior doctors) in the ANZ setting. This paper aims to synthesise the current evidence on effective feedback mechanisms applicable to postgraduate surgical training. Specifically, this review identifies the core principles of effective feedback based on educational theory and empirical research, describes and evaluates established and contemporary feedback models, summarises the evidence regarding the effectiveness of various feedback techniques, discusses common challenges and best practices for feedback delivery in clinical surgical settings, explores the role of simulation and technology in facilitating feedback, and provides recommendations tailored for implementation within the ANZ healthcare system and RACS training programmes, relevant across diverse surgical specialties. The ultimate goal is to provide a resource that supports the continuing education of surgeons and enhances the training experience for the next generation.

Methods/Approach

Literature Search Strategy

To ensure a comprehensive overview of the relevant literature, a systematic search strategy was employed. Major biomedical and educational databases, including PubMed/MEDLINE, Embase, PsycINFO, and ERIC, were searched for peer-reviewed articles published in English. The search focused on publications from the last two decades (approximately 2004-2024) to capture current practices and research, while also including seminal earlier works where relevant. Search terms were combined using Boolean operators and included variations of: (“feedback” OR “performance feedback” OR “educational measurement” OR “assessment”) AND (“surgical education” OR “surgical training” OR “residency” OR “registrar” OR “internship” OR “graduate medical education” OR “operating room” OR “surgery”) AND (“effectiveness” OR “models” OR “techniques” OR “best practices” OR “challenges” OR “simulation” OR “technology”). Specific feedback model names (e.g., “Pendleton”, “SET-GO”, “RIME”, “GROW”) were also used as search terms. Inclusion criteria stipulated that articles must focus on feedback related to performance (technical or non-technical) in postgraduate surgical training contexts. Exclusion criteria included articles focused solely on undergraduate medical education, non-English publications, and studies where feedback was not a primary focus. Reference lists of key articles and relevant reviews were also manually scanned to identify additional pertinent studies.

Evidence Synthesis

Retrieved articles were screened for relevance based on title and abstract, followed by full-text review where necessary. Data extraction focused on identifying: characteristics of effective feedback; descriptions and evaluations of different feedback models; empirical evidence linking feedback interventions to outcomes (e.g., skill improvement measured by assessment tools like objective structured assessment of technical skills (OSATS), changes in behaviour, trainee/supervisor perceptions); documented challenges and barriers to effective feedback in surgical settings; proposed best practices and implementation strategies; the use and impact of technology and simulation in feedback processes; and any findings specifically related to the Australian and New Zealand context. The synthesis aimed to identify recurring themes, compare findings across different studies and contexts, and evaluate the strength of evidence supporting various approaches. This structured approach aligns with the expectation of methodological transparency often valued in surgical research publications.

Framework for Analysis

The analysis and synthesis of the literature were implicitly guided by established principles of adult learning theory and reflective practice [21]. Concepts such as the importance of learner engagement, the need for feedback to be relevant and applicable to practice, the role of reflection in consolidating learning, and the influence of the learning environment on feedback acceptance were considered when interpreting the findings and formulating recommendations [22]. This framework helps ensure that the discussion moves beyond simple description towards an understanding of why certain feedback approaches appear more effective than others. The clear delineation of the methodology allows readers and reviewers to assess the scope and potential limitations of this review, fostering confidence in the subsequent presentation of findings and discussion.

## Review

Results/Key principles of effective feedback

The literature consistently highlights several key characteristics that appear crucial for feedback to be educationally effective in the surgical training context [3,5,23]. These principles provide a foundation for developing optimal feedback practices.

Characteristics of Effective Feedback

Effective feedback is generally described as possessing several essential characteristics [4,6,24]. Timely feedback is delivered as close to the observed performance as feasible, allowing the trainee to readily connect the feedback to the specific event, as delayed feedback loses impact and relevance [25]. Specific and behaviourally focused feedback avoids vague comments such as "good job" or "needs improvement" and instead focuses on specific, observable behaviours or actions, avoiding judgements about personality or inherent ability [5,26]. For example, stating, "Your tissue handling in that step was precise, minimizing trauma" is more effective than saying, "You're good with your hands." Feedback based on direct observation ideally stems from firsthand observation of performance by the supervisor, as relying on hearsay or assumptions undermines credibility and accuracy [27]. Objective feedback is grounded in observed performance against established standards or learning objectives, rather than subjective opinions or biases, and using clear criteria can enhance objectivity [28]. Constructive and balanced feedback, while addressing areas for improvement, often incorporates positive reinforcement for aspects performed well, with the overall tone being supportive and aimed at development rather than criticism [29]. Actionable feedback ensures the trainee understands what they need to do differently to improve and leads to concrete suggestions or a collaborative plan for improvement [30]. Dialogic feedback is most effective when it is part of a conversation, allowing the trainee to ask questions, clarify points, reflect on their own performance, and participate in setting learning goals, rather than being a one-way transmission of information [3,6]. Respectful and considerate feedback is delivered privately and professionally, acknowledging the trainee's perspective and fostering psychological safety [14]. Conversely, feedback perceived as ineffective is often vague, delayed, judgmental, non-specific, based on assumptions, or delivered in a way that feels dismissive or overly critical [15].

Established Feedback Models

Several structured models have been developed to guide feedback conversations, aiming to incorporate the principles described above [3,24]. The Pendleton Model (Pendleton's Rules) follows a specific sequence that includes clarifying any points of fact, having the trainee identify what went well, having the supervisor identify what went well, having the trainee identify areas for improvement, and having the supervisor identify areas for improvement and suggest remedies. Its strength lies in starting with the learner's perspective and reinforcing positive aspects first. However, it can sometimes feel formulaic, and its primary focus is often on analysing a past performance rather than explicitly planning future learning. The SET-GO Model (What I Saw, what Else did I see, what Thinking was involved, what Goals arise, Offers/Opportunities) encourages exploration of the trainee's thought processes behind their actions. The steps involve describing what was seen (specific behaviours), exploring other observations, understanding the trainee's thinking, setting goals for future performance, and identifying offers and opportunities for practice or support. It promotes deeper reflection and collaborative goal-setting. Other frameworks exist, such as the RIME model (Reporter, Interpreter, Manager, Educator), often used for assessing clinical reasoning development, and the GROW model (Goal, Reality, Options, Will/Way forward), adapted from coaching, which focuses strongly on future actions and commitment. While these models provide useful structures, rigid adherence is less important than understanding and applying the underlying principles of effective feedback [3]. The choice of model may depend on the specific context (e.g., post-operative debrief, skills assessment), the learning objective, and the relationship between supervisor and trainee. A comparison of key features is presented in Table 1.

**Table 1:** Comparison of common feedback models in surgical education Table created by authors based on synthesis of literature review findings [3,24,29]. No external reproduction; original table developed for this manuscript. Evidence level is a qualitative summary based on literature review findings. Rigorous comparative effectiveness studies remain limited for many models in surgical contexts. SET-GO: What I Saw, what Else did I see, what Thinking was involved, what Goals arise, Offers/Opportunities; RIME: Reporter, Interpreter, Manager, Educator; GROW: Goal, Reality, Options, Will/Way forward

Feature	Pendleton Model	SET-GO Model	RIME Framework	GROW Model
Key Steps	Clarify; Trainee good points; Supervisor good points; Trainee improvement areas; Supervisor improvement areas & plan	Saw; Else; Thinking; Goals; Offers/Opportunities	Assesses level: Reporter, Interpreter, Manager, Educator	Goal; Reality; Options; Will/Way Forward
Primary Focus	Analysis of past performance; Learner self-assessment first	Understanding cognitive processes; Collaborative goal-setting	Developmental stage of clinical reasoning & responsibility	Future-oriented; Action planning; Coaching approach
Strengths	Learner-centric start; Balances positive/negative	Probes thinking; Promotes reflection; Action-oriented	Provides developmental benchmark for clinical skills	Strongly action-oriented; Empowers learner; Flexible
Weaknesses	Can feel formulaic; May focus less on future plan	Can be time-consuming; Requires skilled facilitation	Primarily evaluative; Less structured for specific event feedback	Less focused on analysing past specific performance
Evidence Level	Widely used, some support but limited comparative trials	Growing evidence, particularly in simulation settings	Established for assessment, less studied as feedback delivery model	Widely used in coaching, less empirical surgical data
Typical Use Case	Post-operative debrief; Procedure review	Complex case discussion; Simulation debrief; Skill refinement	Overall progress assessment; Entrustment decisions	Mentoring sessions; Career development; Problem-solving

Evidence for Effectiveness

While methodologically robust studies directly linking specific feedback models to long-term patient outcomes are scarce, a considerable body of evidence suggests that structured, high-quality feedback positively impacts intermediate outcomes crucial for surgical training [7,8]. Studies utilising validated assessment tools (e.g., OSATS, Global Operative Assessment of Laparoscopic Skills (GOALS)) often demonstrate improved technical skill acquisition when specific, actionable feedback is provided, particularly in simulated environments [1,2,28]. Evidence also appears to support the role of feedback in enhancing non-technical skills, such as communication, teamwork, and decision-making, especially when feedback explicitly addresses these domains [19]. Furthermore, trainees consistently report higher satisfaction and perceive greater educational value when feedback aligns with the principles outlined above (timely, specific, constructive, etc.) [15,23]. Effective feedback practices may also foster better supervisor-trainee relationships and promote a culture of continuous learning and self-reflection [14,22].

Challenges in the surgical environment

Despite the known benefits, translating feedback principles into consistent practice in the complex surgical workplace remains challenging. Key barriers identified in the literature include time constraints, observation quality and quantity issues, supervisor training and variability, trainee factors, and cultural factors [9-13]. The fast-paced nature of surgery, high service demands, and duty hour restrictions often limit opportunities for dedicated feedback conversations before or after procedures [11,12]. Supervisors may not always be present for critical parts of a procedure or may be focused on other tasks, leading to incomplete observation and feedback based on assumptions [27]. Many surgeons receive little formal training in educational theory or feedback techniques, leading to wide variability in the quality, quantity, and style of feedback provided, with some supervisors lacking confidence or feeling uncomfortable delivering constructive criticism [13,16]. Trainees may be hesitant to ask for feedback due to fear of appearing incompetent, perceived lack of supervisor approachability, or feeling overwhelmed, and they may also struggle to process and act upon feedback effectively, particularly if it is poorly delivered [15]. Hierarchical structures, perceived power imbalances, and concerns about interpersonal dynamics can inhibit open and honest feedback exchange, while a culture that prioritises service delivery over education can further marginalise feedback opportunities [14].

Best practices for delivery

Based on the principles of effective feedback and strategies to overcome common challenges, the literature suggests several best practices for supervisors [3,6,24,29]. Supervisors benefit from setting clear expectations by communicating the importance of feedback and establishing a routine for providing and receiving it. Establishing psychological safety creates a learning environment where trainees feel safe to ask questions, admit uncertainty, and receive constructive feedback without fear of humiliation or retribution [14]. Supervisors should make a conscious effort to utilise direct observation by focusing on specific aspects of trainee performance and behaviours [27]. Choosing the right time and place involves providing feedback in a private, quiet setting, free from interruptions whenever possible, recognising that timeliness is key but that immediate feedback in a stressful situation may be less effective than a slightly delayed, more reflective conversation [25]. Using specific language requires focusing on concrete actions and behaviours observed while avoiding generalisations and personal comments [5,26]. Balancing strengths and weaknesses involves acknowledging areas of competence alongside areas needing development [29]. Encouraging self-reflection prompts the trainee to assess their own performance first, as exemplified in the Pendleton model [22]. Supervisors should check for understanding to ensure the trainee comprehends the feedback and its implications by asking clarifying questions. Collaborative goal setting involves working with the trainee to develop specific, achievable learning goals and an action plan based on the feedback [30]. Documenting feedback through brief recording of key feedback points can aid trainee reflection and track progress over time.

Role of technology and simulation

Technology and simulation offer valuable adjuncts to traditional feedback methods, particularly for overcoming time and observation barriers [1,2]. Simulation provides a safe environment for deliberate practice and feedback on technical and non-technical skills without patient risk, and debriefing using structured models (like SET-GO) after simulation scenarios is a well-established effective practice [1,2]. Video review, through recording procedures with appropriate consent, allows for detailed, objective review and feedback at a later time, enabling both supervisor and trainee to analyse performance more thoroughly, which can be particularly useful for identifying subtle technical errors or decision-making patterns [1]. Digital platforms, including electronic logbooks, assessment apps, and dedicated feedback platforms, can facilitate the recording, delivery, and tracking of feedback, potentially improving documentation and accessibility. These tools do not replace face-to-face feedback but can significantly enhance its quality and feasibility.

ANZ-specific considerations

While the core principles of feedback are universal, their application in ANZ requires consideration of the local context. The RACS curriculum's emphasis on competency-based progression necessitates robust assessment and feedback systems [17,20]. Challenges related to providing consistent, high-quality feedback across geographically dispersed training sites (metropolitan, regional, rural) may require specific strategies, potentially involving remote supervision technologies or targeted faculty development [18]. Research originating from ANZ institutions may provide valuable insights into local feedback cultures, successful interventions (e.g., specific supervisor training programmes), or challenges unique to the region, such as feedback across diverse cultural backgrounds of supervisors and trainees. Integrating these local perspectives is vital for developing truly relevant recommendations.

Interpretation of findings

The synthesis of evidence confirms that feedback is a cornerstone of surgical skill development and professional growth, yet its effective implementation remains imperfect [3,7]. The consistent emphasis across studies on characteristics like timeliness, specificity, and actionability likely reflects fundamental principles of learning [4,21]. Timely feedback allows for immediate cognitive links between action and consequence, while specific, behavioural feedback reduces cognitive load by focusing the learner's attention on modifiable actions rather than vague concepts or personal traits [5,26]. The dialogic nature of effective feedback aligns with adult learning principles, recognising trainees as active participants in their own learning who benefit from reflection and collaborative problem-solving [22].

The various feedback models (Pendleton, SET-GO, etc.) offer practical frameworks, but their value lies less in rigid adherence and more in prompting supervisors to structure conversations thoughtfully, ensuring key elements like learner reflection and action planning are addressed [3,24]. The observation that different models may suit different contexts (e.g., SET-GO for complex decision-making, Pendleton for procedural review) suggests that supervisors benefit from a flexible toolkit rather than a single prescribed method. The documented challenges, particularly time pressure and lack of supervisor training, highlight a critical gap between the recognised importance of feedback and the systemic support provided for it within many healthcare institutions [11-13,16].

Implications for ANZ surgical education

These findings have significant implications for surgical training within the RACS framework and the broader ANZ healthcare system. The move towards competency-based training models necessitates more frequent, high-quality, and documented feedback to inform entrustment decisions and guide trainee progression [19,20]. Current RACS assessment tools and processes should be reviewed to ensure they optimally facilitate and integrate meaningful feedback conversations [17].

Addressing the unique challenges of the ANZ context is crucial. For instance, ensuring equitable feedback opportunities for trainees in diverse geographical locations may require innovative approaches, such as structured remote feedback sessions using video technology or enhanced support for supervisors in regional and rural centres [18]. Faculty development programmes focused on practical feedback skills, tailored to the ANZ cultural context and addressing common barriers like time management and delivering difficult feedback, should be a priority for RACS and individual training institutions [13,16]. Promoting a culture of feedback requires more than just training; it necessitates leadership commitment and systemic changes that value education alongside service delivery. Recognising and potentially rewarding educational contributions, including feedback provision, could incentivise engagement. Established ANZ researchers and institutions are well-positioned to lead efforts in evaluating and disseminating best practices suited to the local environment.

Addressing Implementation Barriers

Overcoming the persistent barriers to effective feedback requires a multi-pronged strategy [9,12,13]. Faculty development through mandatory, practical workshops focusing on feedback skills, incorporating role-playing and feedback on supervisors' own feedback techniques, should address time-efficient strategies and managing difficult conversations [13,16]. Structured tools can be integrated into routine workflow (e.g., within operative notes templates, electronic logbooks, or specific feedback apps) to encourage more consistent documentation and delivery. Dedicated time, while challenging to secure, can be explored through brief, dedicated moments for feedback (e.g., a 5-minute debrief immediately post-procedure, scheduled brief feedback sessions) at departmental levels [11]. Leveraging technology through promoting the use of simulation for feedback on specific skills and exploring wider, ethically sound implementation of video recording for performance review can overcome observation limitations [1,2]. Promoting a positive culture involves explicitly discussing the importance of feedback, encouraging trainees to actively seek it, and fostering an environment of psychological safety where mistakes are viewed as learning opportunities, which are key roles for surgical leaders [14]. Peer feedback among trainees can also serve as a valuable adjunct.

The role of institutional culture

Ultimately, individual efforts to improve feedback are necessary but insufficient. Lasting change requires a supportive institutional culture that genuinely values education and professional development [14,16]. This involves resource allocation for faculty development, clear expectations regarding supervisors' educational roles, and systems that facilitate rather than hinder feedback exchange. Leadership at all levels, from heads of department to college executives, must champion feedback as a core component of high-quality surgical practice and training. Creating such a learning environment is not merely an educational goal; it is intrinsically linked to patient safety and the continuous improvement of surgical care [7,8]. While this paper focuses on ANZ, the principles discussed have broad applicability, as the challenges of providing effective feedback in demanding clinical environments are shared across healthcare systems globally.

Limitations

This review has several limitations. The heterogeneity of studies in surgical education feedback makes direct comparison difficult. Many studies rely on self-reported data or intermediate outcomes (e.g., trainee satisfaction, skill scores in simulation), with limited evidence directly linking specific feedback interventions to long-term improvements in clinical performance or patient outcomes. Furthermore, while efforts were made to identify ANZ-specific literature, there may be a relative paucity of high-quality research focusing specifically on feedback practices and challenges within this unique context compared to North American or European settings. The reliance on available snippets also means specific, up-to-the-minute ANZ Journal of Surgery guidelines could not be fully incorporated, necessitating author verification before submission.

Future research directions

Further research is needed, particularly within the ANZ context. Comparative effectiveness studies evaluating different feedback models or delivery strategies (e.g., video-based vs direct verbal feedback) in clinical settings are warranted [1]. Research exploring the impact of cultural factors on feedback perception and effectiveness among diverse trainee and supervisor populations in ANZ would be valuable. Studies investigating the optimal integration of technology, such as feedback apps or video review platforms, into RACS training programmes could provide practical guidance [17]. Developing and validating context-specific tools to assess the quality of feedback conversations or the overall feedback environment within ANZ surgical departments could also drive quality improvement efforts. Collaborative multi-institutional studies, potentially involving leading ANZ researchers and centres, would strengthen the generalizability of findings within the region.

## Conclusions

Effective feedback is indispensable for cultivating surgical expertise and ensuring patient safety. This review synthesises evidence highlighting that optimal feedback is timely, specific, objective, actionable, respectful, and part of an ongoing dialogue. While various models exist, understanding and applying these core principles within a supportive relationship is paramount.

Key recommendations for the ANZ surgical community include prioritising structured faculty development in feedback skills. For all supervisor integrating practical feedback tools and strategies into routine clinical workflow and assessment processes within the RACS framework, leveraging simulation and technology (e.g., video review) to augment feedback opportunities and fostering an institutional culture, led by surgical leaders, that actively promotes psychological safety and values feedback as integral to professional development and high-quality care.

By embracing evidence-based feedback practices, the Australasian surgical community can enhance the training experience, accelerate the development of competent and reflective surgeons, and ultimately, improve patient outcomes. Implementing these strategies represents a crucial investment in the future of surgery in ANZ, and potentially offers valuable lessons for surgical education worldwide.
